# Performance evaluation method for read mapping tool in clinical panel sequencing

**DOI:** 10.1007/s13258-017-0621-9

**Published:** 2017-11-09

**Authors:** Hojun Lee, Ki-Wook Lee, Taeseob Lee, Donghyun Park, Jongsuk Chung, Chung Lee, Woong-Yang Park, Dae-Soon Son

**Affiliations:** 1 Samsung Genome Institute (SGI), Samsung Medical Center (SMC), Seoul, 06351 South Korea; 2 Department of Digital Health, SAIHST, Sungkyunkwan University, Seoul, 06351 South Korea; 3 Department of Molecular Cell Biology, Sungkyunkwan University School of Medicine, Suwon, 16419 South Korea; 4 Department of Health Sciences and Technology, SAIHST, Sungkyunkwan University, Seoul, 06351 South Korea

**Keywords:** Next-Generation Sequencing, Clinical panel sequencing, Alignment tool, Read mapping, CancerSCAN

## Abstract

In addition to the rapid advancement in Next-Generation Sequencing (NGS) technology, clinical panel sequencing is being used increasingly in clinical studies and tests. However, tools that are used in NGS data analysis have not been comparatively evaluated in performance for panel sequencing. This study aimed to evaluate the tools used in the alignment process, the first procedure in bioinformatics analysis, by comparing tools that have been widely used with ones that have been introduced recently. With the accumulated panel sequencing data, detected variant lists were cataloged and inserted into simulated reads produced from the reference genome (h19). The amount of unmapped reads and misaligned reads, mapping quality distribution, and runtime were measured as standards for comparison. As the most widely used tools, Bowtie2 and BWA–MEM each showed explicit performance with AUC of 0.9984 and 0.9970 respectively. Kart, maintaining superior runtime and less number of misaligned read, also similarly possessed high level of AUC (0.9723). Such selection and optimization method of tools appropriate for panel sequencing can be utilized for fields requiring error minimization, such as clinical application and liquid biopsy studies.

**Electronic supplementary material:**

The online version of this article (doi:10.1007/s13258-017-0621-9) contains supplementary material, which is available to authorized users.

## Introduction

After the emergence of Sanger sequencing 40 years ago, more precise, massive-running, and rapid methods of aligning reads to a huge reference genome have been developing consistently. Innovative technology such as Next-Generation Sequencing (NGS) is widely used in genetic variant detection and has successfully transited from bench-to-bedside in the recent decade. NGS platforms, such as MiSeqDx and Oncomine Dx, have already received approval from the US Food and Drug Administration (FDA) for in vitro diagnostic tests and numerous hospitals are already utilizing these systems for clinical trials. Furthermore, NGS technology is also starting to become commercialized; for instance, FoundationOne (https://www.foundationmedicine.com/) and Personal Genome Service Genetic Health Risk (https://www.23andme.com/) provide doctors or clinicians the necessary biomarkers of the potential patient for clinical trials or targeted therapies.

From the advancement in technology, the price of sequencing and the turnaround time has decreased rapidly and clinical application is thus becoming more accessible. NGS in clinical diagnostics actively makes use of targeted panel sequencing, a method that targets only a small portion of the whole genome, mainly by detecting actionable mutations using panels comprising known hotspots (Cheng et al. 2015; Easton et al. 2015; Kurian et al. 2014). In South Korea, specifically, gene panel testing based on NGS has been covered by the National Health Insurance since March 2017. Therefore, acceleration of panel tests on patients can be readily anticipated. A clinical gene panel test based on NGS involves numerous complicated steps that need optimization and quality management. Specifically, the detailed processing of bioinformatics analysis such as the read mapping step, deduplication, local realignment, and variant calling process, is gaining more importance. For each step, various algorithms have been developed and their performances are compared with each other. Consistent performance evaluation with quality control is imperative for clinical examinations, as such analysis influences the diagnostics and treatments in actual patients.

Since clinical panel sequencing is primarily aimed for clinical analysis, it requires an optimization procedure with a different standard from that of whole genome or whole exome sequencing. Clinical panel studies vary in the location of hotspots to be targeted and such data can be influenced by the specimen type, library manufacturing protocol, and sequencing condition. Furthermore, mutations can differ by race and disease type, and therefore, selecting an optimal tool and parameter for such variations in data is a very crucial procedure for precision medicine.

The Samsung Genome Institute (http://sgi.samsunghospital.com/) has developed and utilized CancerSCAN, based on the data from more than 10,000 panel sequencing studies since 2014 (Kim et al. 2017). With the accumulated data, the general pipeline has been optimized for the CancerSCAN panel and this study will focus on the alignment tool, which is the first step in the analysis process.

The first step in the analysis of NGS data is alignment, i.e., specifically aligning the generated reads to the human reference genome sequence to locate the chromosomal position. The aligned positions become the basis for variant detection analysis and therefore, faulty alignments or systematic biases lead directly to variant detection errors. Numerous aligners have been proposed so far with distinguishing properties in order to achieve greater accuracy pertaining to precision. Previous studies have reviewed the tools according to mapping strategy (Bao et al. 2011; Fonseca et al. 2012), tool characteristics, input data type (Thankaswamy-Kosalai et al. 2017), and manipulating the tool parameter options (Hatem et al. 2013). The Hatem group specifically compared nine tools with RNA-seq data and manually optimized the parameters, changing the number of allowed mismatches and gapped lengths, to achieve a reduced runtime while sacrificing the quality of the result. The runtime of algorithms is also regarded as an important parameter in the aspect of turnaround time in clinical panel sequencing. Although library preparation and sequencing is the more time-consuming process, excessively longer NGS gene panel tests compared to other clinical tests, is a problem that needs to be solved.

Widely used tools with superior performances in previous studies (Thankaswamy-Kosalai et al. 2017) were initially evaluated including Bowtie 2 (Langmead et al. 2009), BWA–MEM (Li and Durbin 2009), and NovoAlign (http://www.novocraft.com). Furthermore, tools that were developed after the previous review papers or those that have not been widely compared previously in review papers, were also selected and included BatAlign (Lim et al. 2015) showing higher sensitivity and specificity than BWA–MEM and Bowtie 2 when processing short reads with structural variants, BWA–PSSM (Kerpedjiev et al. 2014) outperforming the sensitivity of BWA–MEM and Bowtie 2 using position-specific scoring matrices for high divergent reads such as ancient DNA, CUSHAW3 (Liu et al. 2014) scoring higher sensitivity compared to BWA–MEM, Bowtie 2 and NovoAlign for short reads, Kart (Lin and Hsu 2017) reducing the runtime to about 3–100 times compared to BWA–MEM and Bowtie 2 with both short and long reads, and NextGenMap (Sedlazeck et al. 2013) showing shorter runtime and higher percentage of correctly mapped reads compared to BWA and Bowtie 2 when processing high divergence reads.

Panel sequencing also differs from whole genome or whole exome sequencing pertaining to mapping performance. The reason for such discrepancy is that (1) excessive time can be wasted on processing a portion of the genome without hotspots, since clinical tests usually focus on hotspots of interest and (2) the mutation type and length to be detected can largely influence performance. BatAlign specifically, can accurately detect fusion reads such as structural variants and polymorphic-variants (Lim et al. 2015). Also (3) multiple alignments, or secondary alignments, on comparatively narrow target regions can show a more simplified determination of detection compared to whole genome sequencing. Therefore, panel sequencing requires an optimized analytical pipeline that can maximize its performance regarding the various properties it possesses.

This study aimed to organize the single nucleotide variants (SNVs) and insertion/deletions (InDels) that occur in Koreans based on the 8378 analyzed results data from CancerSCAN. In addition, using the CancerSCAN data, we can seek an appropriate alignment tool for CancerSCAN. Although the algorithms for each tool possess different strengths and features, this study will emphasize on comparing how many reads were mapped in their correct positions. If the reads were to be mapped in a different position, the wrongly mapped positions can be detected as variants, and therefore become false positives, which is critical in clinical tests. Hence, the proportion of misaligned and unmapped reads is the most important measure, since it can mislead the result of alignment and clinical decisions. In this study, we compared the performance of aligners by evaluating the number of aligned, misaligned, and unmapped reads using simulated reads. The reads were generated from the Human reference sequence using in silico simulation and the original position in which the reads were generated from was compared with the mapped position resulting from each tool alignment.

## Materials and methods

### In silico read generation

In silico read generation was based on the human reference genome (hg19). However, among various read simulators, none possessed the ability to identify the location from which the reads were generated, insert the variants with the desired variant allele frequency, and determine the target region to be sequenced for panel sequencing. Therefore, DWGSIM (http://github.com/nh13/dwgsim) was only used to report the read generated position and FASTQ datasets with variants were produced according to the following methods.

#### Step I: simulated read data generation

The reference genome sequence was modified to generate reads within the target area for panel sequencing. The areas ± 200 bp from each target obtained from CancerSCAN were put together to reconstruct the reference. With the reference prepared, DWGSIM was used to simulate pair-ended reads with a read length of 100 bp. As input parameters, the base error rate was set as 8.25E−05 and the insert size was 175 ± 51.85 (mean ± SD, bp) obtained from a mix of 10 HapMap cell lines from a previous study (Chung et al. 2016). The read production was 4Gbase with a mean coverage of 1140×, equated with that of CancerSCAN. All generated reads were assessed to determine whether they were correctly produced from the corresponding target region. Reads that overlapped between different target regions were excluded.

#### Step II: variant insertion

Variants were inserted into the FASTQ files generated in *Step I*. Variants were selected from 40,602 SNVs and 198 InDels (Supplementary Table 1) which were called from 8378 cancer patient samples in CancerSCAN. The number of variants to be inserted was chosen according to the listed eight sets in Table 1. All reads covering each variant position were modified according to the corresponding variant allele frequency obtained from CancerSCAN. Maximum length of each insertion and deletion was 25 and 30 bp, respectively.

**Table 1:** Number of SNVs and InDels inserted in simulated FASTQ sets

Variant type	Set 1	Set 2	Set 3	Set 4	Set 5	Set 6	Set 7	Set 8
SNV	0	20	40	80	160	230	300	4111
Insertion	0	1	5	10	20	35	48	48
Deletion	0	2	5	10	20	35	53	53

Therefore, in the *i*th FASTQ file, if the read depth of the *j*th variant position is *n*_*ij*_, the variant was inserted according to the variant allele frequency of the corresponding variant *p*_*ij*_ (Supplementary Table 1). Thus, if the number of reads that possesses variants in the *j*th position is the random variable *X*_*ij*_, the distribution of *X*_*ij*_ will resemble a binomial distribution as such.

$${X_{ij}}~\sim ~B~\left( {{n_{ij}},~{p_{ij}}} \right)$$

In total, 8 FASTQ file sets were produced, ranging from “Set 1” containing no variants to “Set 8” containing more than 4000 variants. Each set was produced three times repeatedly (Table 1). Variants were inserted from a random selection among the variant lists from Supplementary Table 1 and FASTQ files within the same sets had the same variant type and matching variant allele frequency.

### Alignment tool selection and features

Fonseca et al. (2012) reviewed the characteristics of 60 alignment tools and compared functions such as maximum and minimum read length, number of allowed mismatches, etc. Referring to the various features of each tool, tools were appropriately selected for the given server and data type to be handled. The standards of selection were based on accessibility (open source material), alignment option (DNA-based paired-end sequencing allowing mismatches, InDels, gaps) and the quality awareness of the tools (utilization of mapping quality). Tools that were not compatible with our system, AlignerBoost (Zheng and Grice 2016) and CORA (Yorukoglu et al. 2016), were excluded from the list.

The eight selected tools and their basic features are listed in Table 2. The number of citations was obtained from Web of Science (https://webofknowledge.com/) on September 28, 2017. BWA–MEM has been the most cited followed by Bowtie 2. The recently published tool, Kart and the unpublished tool, NovoAlign, had no citations. More than half the tools were published before 2014, and Bowtie 2 and BWA–MEM were used popularly despite the existence of new tools. Tools that specified the exact number of mismatches, InDels, and gaps in Table 2 were marked with the corresponding numbers, and tools that used the score function were indicated with “score” in the column. The mapping quality range used in evaluating the reads was also sorted in the table. While BatAlign, BWA–MEM, Kart, and NextGenMap use a range from 0 to 60, other tools used different mapping quality ranges. The factors common to all eight tools were that they have FASTQ as the input format and SAM as the output format. However, BWA–PSSM preprocesses each FASTQ file to SAI file and then the two SAI files from each paired-end read are combined to form a single SAM file.

**Table 2:** List of alignment tool and features

Alignment tool	Version^a^	Citation^b^	Published year	Citations/year	Mismatch	InDels	Gaps	MQ range
BatAlign	v1	3	2015	1.5	5	Y	200	0–60
Bowtie 2	v2.3.2	7227	2009	843.6	Score	Score	Y	0–42
BWA-MEM	v0.7.15	8494	2013	1035.5	Y	8	Y	0–60
BWA-PSSM	v0.7.8	15	2014	4.3	Y	8	Y	0–200
CUSHAW3	v3.0.3	177	2014	48.1	Y	Y	Y	0–250
Kart	v2.2.1	0	2017	0	Y	5	5	0–60
NextGenMap	v0.5.0	55	2013	14.1	Score	Score	Y	0–60
NovoAlign^c^	v3.08.00	–	2014	–	8	7	Y	0–70

Mismatch and InDels column shows the number of mismatches and InDels allowed in the alignment by default. Score indicates that the mapper uses score function. Gap column shows if consecutive InDels are permitted in alignment, as if possible the length of gaps in base pair. Yes is abbreviated as Y

^a^Version: the tool versions used were the latest versions as of August 23, 2017

^b^Citation: the number of citations of tool publications was obtained from Web of Science on September 28, 2017

^c^NovoAlign: NovoAlign is not published and can be accessed through http://www.novocraft.com. The published year for NovoAlign is the year of its first version

### Method of evaluation

Evaluation of alignments was performed using the original chromosomal position for each pair of generated reads via the DWGSIM simulator. The original chromosomal positions were separately and distinctively stored in a separate file and were compared with the starting position of the mapped read after alignments of the tools. If the starting positions of the mapped read did not exactly match the respective separately stored original position or the position that the read was actually generated from, the read was considered as wrongly mapped and therefore a misaligned read. Even if the reads were mapped in different locations because of sequence homology, they were considered as misaligned. Alignments were accepted as correct if and only if the starting positions agreed exactly, despite any existence of variants in the reads or any other sequence errors. Every read was evaluated using such methods to examine the mapping performance of the tools.

Other factors such as time were also used as an evaluation standard. Of the eight tools, seven were run under four threads, CentOS 6.6, and RAM 192 GB with CPU 2.40 GHz (12 cores); NovoAlign proceeded under a single thread with the same RAM and CPU because the multi-threaded option was only available commercially. The time required for the alignment process was measured until a final SAM file was made.

The mapping quality for all tools had a cutoff value of zero. In other words, reads were not filtered out even if the mapping quality of a specific read was very low. Additionally, Bowtie 2 (mapping quality range of 0–42), BWA–PSSM (0–200), CUSHAW3 (0–250), and NovoAlign (0–70) had different calculation methods for mapping quality; the scores were linearly transformed to fit the range of 0–60 like any of the other tools. Therefore, we could visualize every read without filtering, and determine any tendencies in misalignments for each tool.

## Results

### Number of simulated reads

Reads were produced with a coverage of 1140× in each set, rather than exactly matching the total number of reads. Therefore, the average number of reads in a set was 66,894,525 reads with a standard deviation of 5616 reads which is approximately 0.8% of the average (Supplementary Table 2).

### Repeatability

The repeatability of each alignment tool was confirmed by a reiterating alignment for Set 1 and Set 8. Tools other than BatAlign produced the exact same alignment result for all reads, whereas BatAlign had a different alignment result for the two trials. The total numbers of misaligned reads in the two trials were fairly the same; however, roughly 1% of the reads was initially mapped properly, read in one trial, and misaligned in the other, creating a discrepancy in repeatability (Supplementary Table 3).

### Misaligned and unmapped reads

**Fig. 1:**
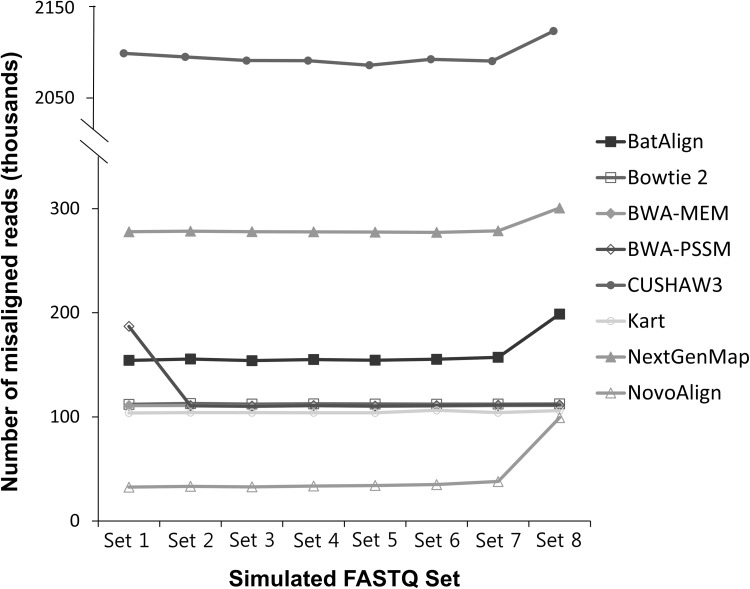
Number of misaligned reads for each simulated FASTQ set. The average number of misaligned reads obtained by comparing the alignment result with the original position of the reads. The detailed average and standard deviation are listed in Supplementary Table 4

All simulated reads, Sets 1–8, were mapped to the whole hg19 reference sequence. Since the reads were simulated in silico from the reference genome, alignment results were expected to be nearly perfect. However, CUSHAW3 resulted with average of 2,099,439 misaligned reads, 3% of total reads, in Set 1 and even increased to 2,124,189 misaligned reads, 3.1% of total, in Set 8, which carried the most variants (Fig. 1). Misaligned reads, unlike unmapped reads, are regarded as more misleading, since they produce false positive results from mapping to the wrong location and are reported as mutation supporting reads. Tools other than CUSHAW3 had a similar level of average number of misaligned reads, with NovoAlign possessing the lowest. The number of misaligned reads in the tools was overall insensitive to the number of variants in the set. Exceptionally, in BatAlign, NextGenMap, and NovoAlign, misaligned reads increased for Set 8, when an excessive amount of variants were present. In contrast, BWA–PSSM had average of 186,931 misaligned reads, 3% of total reads, in Set 1, while sets with variants showed a lower proportion of misaligned reads at 2%, which is approximately 110,762 misaligned reads per sets.

On the other hand, for the average number of unmapped reads, NovoAlign performed with 21,071 unmapped reads, 0.3% of total, even in simulated read sets without any inserted mutation like Set 1, and 161,209 reads were unmapped in NextGenMap, 0.2% of total (Supplementary Table 4). Kart and BWA–PSSM also showed unmapped reads in Set 1, though very small. The proportions and average number of unmapped reads in the above tools neither increased nor showed any such tendency as the number of inserted variants increased from Sets 1 and 8. The unmapped reads in Bowtie 2 appeared only when the number of variants increased in Sets 7 and 8. Other tools like BatAlign, BWA–MEM, and CUSHAW3 did not have any unmapped reads in all repetitions of the simulated sample sets.

### Mapping quality distribution

**Fig. 2:**
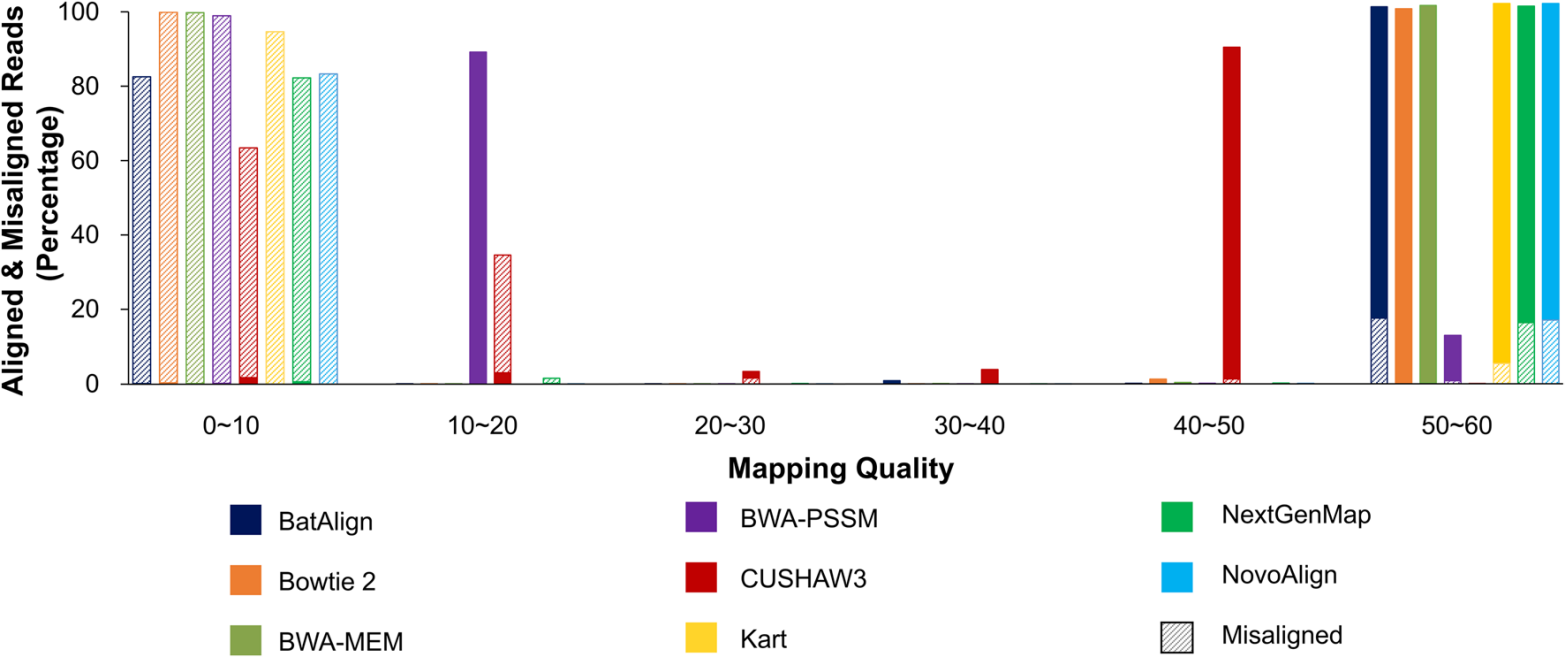
Mapping quality distribution for aligned and misaligned reads. Mapping quality for aligned and misaligned reads was calculated and the reads were grouped into six categories according to their scores. The solid bars indicate the properly aligned reads and the dashed boxes indicate the misaligned reads. The mapping quality range for all tools was equalized from 0 to 60 using linear transformation

**Fig. 3:**
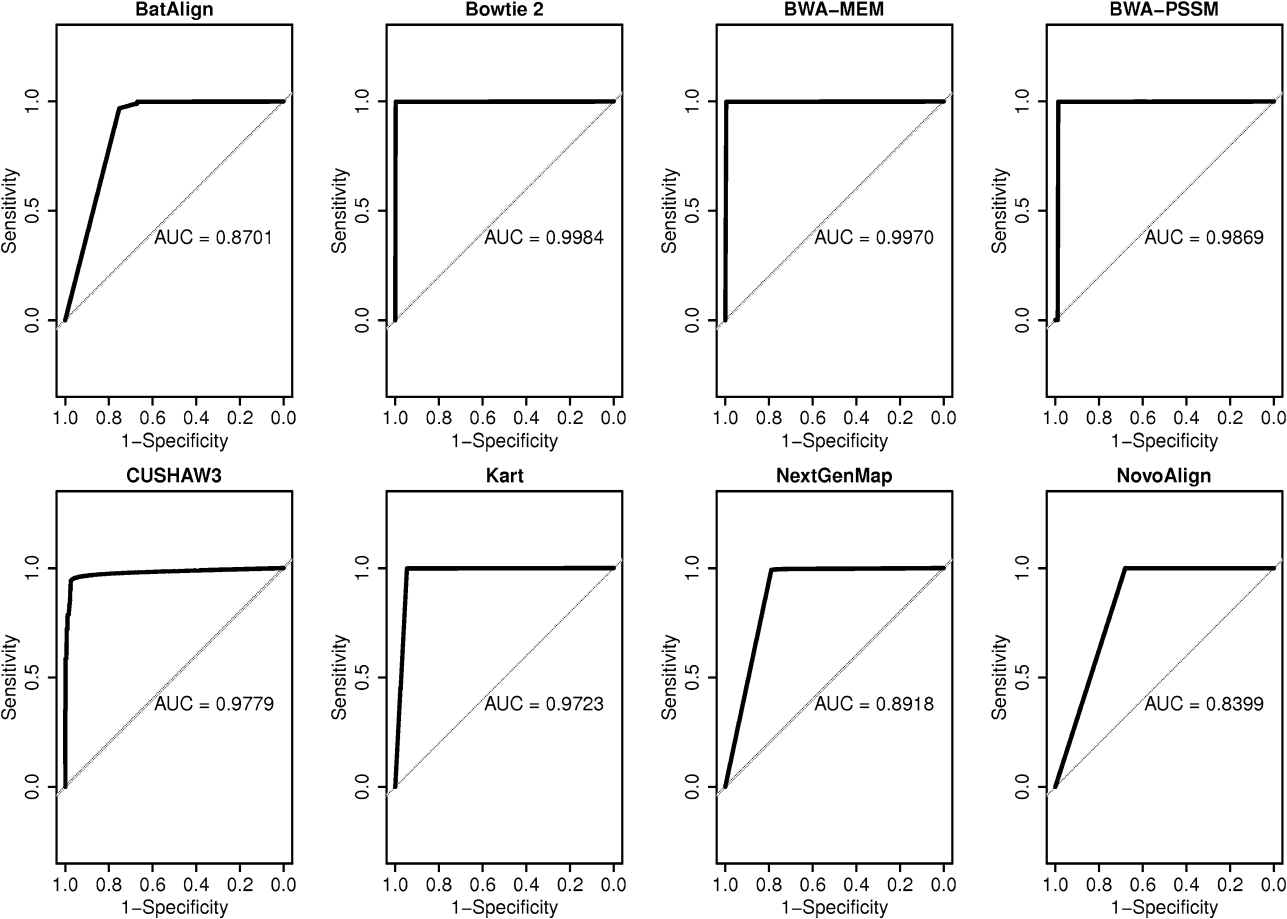
ROC analysis of read mapping quality in Set 8. ROC curve and the corresponding AUC was displayed for the mapping quality result on the alignment result of Set 8 for each tool

The mapping quality differences for each tool, between properly aligned reads and misaligned reads are compared in the graph (Fig. 2). Such distinction is fundamental when setting a threshold to filter the misaligned reads from properly aligned reads. The mapping quality distribution for properly mapped reads in BWA–MEM is concentrated at the highest group of mapping quality; more than 99% of properly mapped reads possessed a mapping quality of over 50. Similarly, for misaligned reads, 99.8% of the reads were distributed in the lowest group of mapping quality, proving that BWA–MEM is capable of effectively separating and removing the misaligned reads during alignment. Bowtie 2 showed a similar performance and thus, is also confirmed to be an effective tool to discard misaligned reads. The distribution in BWA–PSSM, in contrast to the other seven tools, had a very low percentage of properly aligned reads at the highest group of mapping quality. Such a phenomenon might have occurred since BWA–PSSM initially reports mapping quality with a range from 0 to 200, and the process in which we linearly transformed the scores could have been misleading. Nevertheless, the fact that the ability to discern misaligned reads using BWA–PSSM was inferior to that of BWA–MEM could be affirmed. BatAlign, NovoAlign, and NextGenMap had approximately 15% of misaligned reads scored at the highest group of mapping quality, thus failing to show effective distinguishing abilities on the reads. Receiver operating characteristic (ROC) analysis on the mapping quality was performed on alignment result of Set 8, including the most variants. As previously mentioned, Bowtie2 and BWA–MEM each showed the highest class of AUC, 0.9984 and 0.9970 respectively, followed by BWA–PSSM, CUSHAW3 and Kart, in order (Fig. 3).

### Runtime

**Fig. 4:**
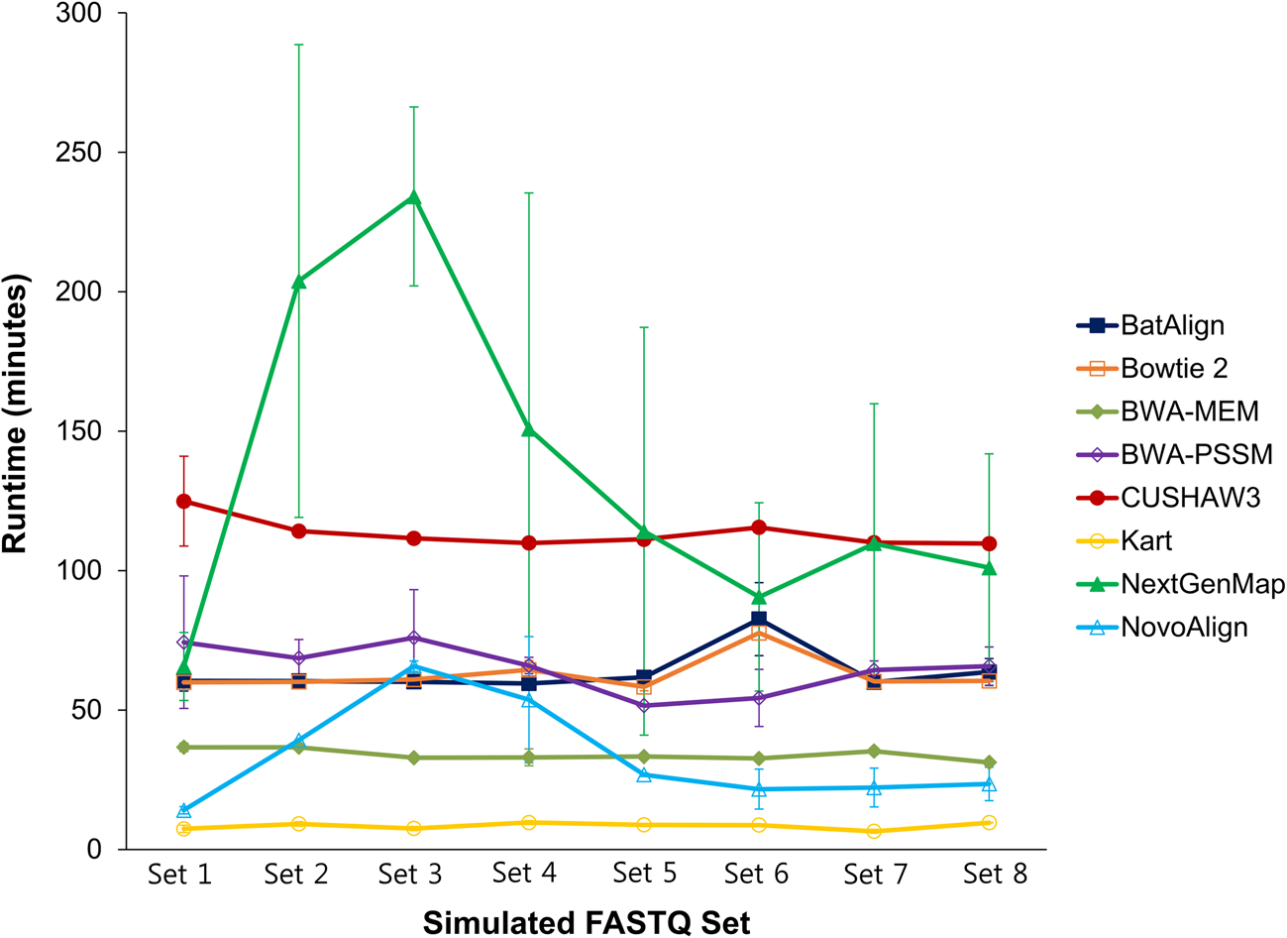
Runtime of tools for different simulated FASTQ sets. Each set had three repetitions and used four threads when aligning the reads. NovoAlign was the only tool that used a single thread

The runtime for each tool was measured from the execution of tool command until the SAM output was generated (Fig. 4). Severe fluctuation in time was observed in NextGenMap and the standard deviation in each set was the highest. Kart maintained the fastest runtime for all sets, followed by NovoAlign, BWA–MEM, Bowtie 2, BatAlign, and BWA–PSSM, in that order, and all finished within 1 h. Among those tools, NovoAlign varied in performance time between sets, whereas Kart and BWA–MEM were very stable in time for different numbers of mutation.

## Discussion

BWA–MEM, as the most popular tool, had no drawback in its performance. Kart showed excellent performance in runtime and a low misaligned read proportion; however, 5.3% of the misaligned reads had high mapping quality thus causing a difficulty separating the mapped and misaligned reads properly, and unmapped reads were also present. NovoAlign had the least percentage of misaligned reads with a very short alignment runtime, despite only a single thread being used, but also had the highest unmapped read percentage and problems in filtering with roughly 16% misaligned reads showing mapping quality.

Most of the misaligned reads were related to the X or Y chromosomes. Specifically, half of the misaligned reads occurred between the two sex chromosomes; reads produced from the X chromosome were mapped to the Y chromosome and the reverse also occurred. Such tendency most likely occurred because the sequence homology between the X and Y chromosomes was comparatively higher than other chromosomes. As to specify the cause of misaligned reads, the sequence of the misaligned reads was further analyzed. Thus, the misaligned reads of Set 1, having identical sequence to the reference since no variants are inserted, was examined to determine whether the misaligned reads are based on the reference sequence homology (Table 3). Of the total misaligned reads, ranging from 37 to 99% for different alignment tools was aligned to locations with different position and sequence. As such reads were misaligned despite the high similarity to the original position of each read, misaligned reads in the presence of perfectly identical sequence can be considered as not directly related to the performance of the tools. However, misaligned reads in nonidentical sequences should be regarded as non-reasonable misaligned reads. Kart possessed the least non-reasonable misaligned reads, 37.1% of total misaligned reads, along with the total number of misaligned reads. Although misaligned reads only take small proportion of the total reads, such misaligned reads that are non-reasonable are most likely to lead to false positive variants.

**Table 3:** Characteristics of misaligned reads in Set 1

Set 1	Count of misaligned reads			Percentage of misaligned read		
	Total misaligned	Identical sequence	Different sequence	Total misaligned (%)	Identical sequence (%)	Different sequence (%)
BatAlign	154,232 ± 348.7	65041 ± 304.3	89,191 ± 47.1	100.00	42.2 ± 0.1	57.8 ± 0.1
Bowtie 2	112,071 ± 278.9	65237 ± 361.1	46,834 ± 177.6	100.00	58.2 ± 0.2	41.8 ± 0.2
BWA-MEM	110,809 ± 374.6	64787 ± 124.8	46,022 ± 331.5	100.00	58.5 ± 0.2	41.5 ± 0.2
BWA-PSSM	186,931 ± 737.5	34672 ± 157.6	152,259 ± 813.7	100.00	18.5 ± 0.1	81.5 ± 0.1
CUSHAW3	2,099,439 ± 3718.2	139615 ± 44.6	1,959,824 ± 3732.9	100.00	6.7 ± 0.0	93.3 ± 0.0
Kart	103,813 ± 46	65261 ± 113.1	38,552 ± 137.6	100.00	62.9 ± 0.1	37.1 ± 0.1
NextGenMap	277,783 ± 315.2	63498 ± 80.4	214,285 ± 306.3	100.00	22.9 ± 0.0	77.1 ± 0.0
NovoAlign	32,455 ± 149.8	104 ± 19	32,351 ± 158	100.00	0.3 ± 0.1	99.7 ± 0.1

Misaligned reads are classified as identical sequence when the read sequence is identical to the reference sequence of the aligned position. If the two sequences are different, such misaligned reads are classified as different sequence

The counts of misaligned reads were repeated three times and the data shows the mean ± standard deviation

Additionally, 10% of misaligned reads were mapped out of the targeted region. As currently used tools do not utilize the target region for alignments, alignments of reads to the reference genome have high probability of being mapped off the target region. If tools are to be developed to align reads based on the user-defined target region, the tools would not only decrease the runtime, but also decrease the misaligned reads by effectively mapping reads, formed from secondary alignments on locations with high sequence homology, to the correct position, and will therefore directly relate to increasing the precision of clinical panel sequencing.

Unmapped reads are discarded after sequencing and could result in financial loss. Misaligned reads, on the contrary, are mapped to wrong positions and could further lead to results with variants that do not exist or dilute the support reads for existing variants. Such false positives and false negatives are negligibly small in situations with a plethora of reads and can be trivial to the result, but they can cause significant problems in cfDNA sequencing, a recently introduced technology. Sequencing for liquid biopsy samples requires an ultra-sensitive method that can detect variants even with an allele frequency under 0.1%. Therefore, sequence errors, measured from normal sample data, can be used for modification (Newman et al. 2016) and such a sequence error rate is reported differently for each substation class (Chung et al. 2016; Newman et al. 2014; Park et al. 2017). Moreover, various efforts are made to minimize and compensate the error in bioinformatics processes, such as the development of a Unique Molecule Identifier, which can separate the significant signals mixed with PCR duplicates (Gregory et al. 2016; Schmitt et al. 2012, 2015). As the application of NGS advances, lowering the error level that occurs stochastically will be vital in improving detection sensitivity.

Based on this study, we plan to optimize the tool parameters for post-alignment procedures, which include deduplication and local realignment processes. Furthermore, diverse detection caller algorithms are used for different variant types and their purposes, since the claimed performances vary with respect to the variant allele frequency and limit of detection. Therefore, selecting an appropriate detection caller regarding the purpose of the panel and target variant is compelling. As a beginning for such steps, we have attempted to select the most efficient and suitable alignment tool for CancerSCAN. We further expect that the proposed in silico simulation and performance evaluation methods will contribute to the development of novel panels for laboratories with a similar purpose.

## Electronic supplementary material

Below is the link to the electronic supplementary material.

Supplementary material 1 (XLSX 37 KB)
